# The impact of abandoned iron ore on the endophytic bacterial communities and functions in the root systems of three major crops in the local area

**DOI:** 10.3389/fmicb.2025.1536083

**Published:** 2025-01-21

**Authors:** Shuyi Chen, Jie Tang, Junqiang Xu, Lianxin Peng, Peng Wu, Qiang Li

**Affiliations:** ^1^Key Laboratory of Coarse Cereal Processing, Ministry of Agriculture and Rural Affairs, Sichuan Engineering and Technology Research Center of Coarse Cereal Industrialization, School of Food and Biological Engineering, Chengdu University, Chengdu, China; ^2^Yunnan Plateau Characteristic Agricultural Industry Research Institute, Yunnan Agricultural University, Kunming, China

**Keywords:** root soil, endophytic bacterial diversity, microbial community, mining area crops, ecological restoration

## Abstract

**Introduction:**

Global mining activities have significant impacts on ecosystems, but most studies have focused only on the relationship between soil physicochemical properties and microbial diversity in soils. The present study provides an insight into the effects of mining activities on soil physico-chemical properties and endophytic bacterial community composition in the rhizosphere of three different crops.

**Methods:**

*Musa basjoo Siebold* L., *Amygdalus persica* L., and *Triticum aestivum* L. were collected from the inter-root soils and plant roots to determine the soil physicochemical properties and endophytic bacterial communities in the root system.

**Results:**

The results showed that mining resulted in soil acidification, altered trace element content and increased organic carbon. There was an increase in the *Ascomycota* and *Actinobacteria* phylum of crop root bacteria. Interestingly, the chao1 and shannon indices of the root endophytes of the mining crop were significantly elevated compared to the contro (*p* < 0.05). Among them, *Musa basjoo Siebold* showed the highest level of community richness in the mining environment. The mining environment resulted in functional enrichment of histidine kinases and oxidoreductases in the bacterial community. The total potassium (TK) content in the soil, as well as the Fe and Pb content, were positively correlated with the α-diversity index and *Streptomyces*. Zn and Ti content were significantly negatively correlated with the α-diversity index.

**Discussion:**

This study provides data support for exploring the mechanisms of plant response to the mining environment and developing ecological restoration strategies for mining areas.

## Introduction

Against the background of rapid development in the global industry, the exploitation of mineral resources has become an important cornerstone in supporting social and economic construction. However, this process is commonly linked with substantial shifts in the ecological environment, resulting in a particularly important effect on soil ecosystems (Yuan et al., 2023; Back et al., 2024). In Ohio, United States, Deep ripping by mining increases hydraulic conductivity and reduces soil capacity hindering reforestation, these changes act on the soil, profoundly remodeling its physical and chemical characteristics (Nwovu et al., 2021; Ran et al., 2023). Among the many environmental challenges, heavy metal pollution is characterized by its widespread distribution, strong concealment, difficulty in removal, and far-reaching harm. Furthermore, heavy metal pollution can enter organisms through absorption by plant roots, gradually accumulate, and be transmitted along the food chain, ultimately posing a threat to human health (Liu et al., 2023b; Badamasi et al., 2024; Yu et al., 2024). Therefore, heavy metal pollution in the soil and crops growing in mining areas has attracted the close attention and in-depth exploration of scientific researchers (Xiang et al., 2021; Dong et al., 2023). Research has demonstrated that nutrients and heavy metals present in abandoned tailings surfaces are redistributed during intense erosion, leading to the destabilization of nearby ecosystems and impacting human health. Vegetation management has been shown to enhance mine soils, ultimately improving the ecological safety of tailings by boosting soil aggregate stability, soil organic carbon levels, and other key components (Ju et al., 2024). Yunnan, situated in Southwest China, boasts abundant mineral resources and a diverse range of wildlife. It is also an important agricultural production area. Soil is the basis of agricultural production, and changes in its physicochemical properties directly affect the growth, development, yield, and quality of crops. These changes can potentially harm food safety and human health in the long term, as they may transfer through the food chain (Kumar et al., 2022; Liu X. et al., 2023).

Endophytes are present in almost all plant tissues, usually in vascular tissues, and then spread throughout the entire plant (Nunna and Balachandar, 2024). Plant roots are essential for the regeneration of mineral nutrients and the removal of harmful toxins in the soil. They also contribute to the decomposition of organic matter and the maintenance of soil structure, forming a sophisticated ecological network (Blagodatskaya and Kuzyakov, 2013). Endophytes not only obtain necessary nutrients from the host plant but also contribute to the improvement of plant growth and development by impacting the physiological metabolic processes of the host (Noman et al., 2021; Ali et al., 2024). They produce and release phytohormones, increasing the stress resistance of plants. They effectively defend against the invasion of alien pathogens and maintain the health of plants (Li M. et al., 2022; Leng et al., 2023; Ran et al., 2024). The plant microbiome composition is constantly changing due to various abiotic and biotic factors. The structure and function of microbial communities can be influenced by the bacterial communities in the soil, which are greatly shaped by environmental conditions (Yang et al., 2020; Ahmed et al., 2024; Li W. et al., 2024). Consequently, a detailed understanding and precise scientific control of these influencing factors are necessary for optimizing soil ecosystem structure and boosting agricultural production efficiency.

Numerous research studies have explored the impact of soil condition alterations on plant microbes. VTM (vanadium and titanium magnetite) mining leads to soil acidification and metal contamination, posing challenges for nearby agricultural lands. Despite this, tobacco cultivation has been found to enhance soil enzyme activities and facilitate the growth of resilient bacteria (Fu et al., 2024). The combination of a high nutrient supply and a diverse plant species can have a beneficial impact on soil microbial communities, potentially mitigating the harmful effects of metal pollution (Stefanowicz et al., 2012). In terms of soil improvement, the addition of mineral-microbe composites to the matrix has become an effective means. This not only promotes plant growth and increases the fertility index of the matrix, but also significantly increases the abundance and diversity of biological communities (Zhang et al., 2022). Moreover, by restoring vegetation, the Pb content in the soils of Pb-Zn tailings production areas was effectively reduced, leading to a shift in the dominant genus composition of the soil microbial communities. This highlights the crucial role of vegetation in remediating heavy metal-contaminated soils (Wen et al., 2024). Studies have shown that an increase in the soil available phosphorus content has a positive effect on the growth environment of corn and significantly improves the diversity of soil microbes (Campolino et al., 2023). As a key environmental factor, soil organic carbon (SOC) has a profound effect on the distribution and structure of root bacterial communities, endophytes and fungi, whereas carbonate ions (CO₃^2−^) have a more specific regulatory effect on root fungal communities (Luo et al., 2023). However, the complex interaction modes between environmental factors and the structural diversity of the root microbial community remain unclear (Chen et al., 2020).

*Musa basjoo Siebold* L. is a significant fruit in tropical regions, with its pulp containing essential nutrients like sugars, amino acids, and dietary fiber. These nutrients not only provide the required energy and building blocks for the human body but also offer potential medicinal benefits. Researchers highly value this fruit for its nutritional and medicinal properties (Pereira and Maraschin, 2015; Wei et al., 2019). *Triticum aestivum* L., an indispensable staple crop, is rich in energy and plays a vital role in the maintenance of body functions and the promotion of health (Shewry and Hey, 2015; Rasheed et al., 2024). *Amygdalus persica* L. has a delicious flesh that is rich in vitamin groups, minerals and antioxidants. These nutrients have been shown to be effective in strengthening the body’s immune system and protecting it from disease (Shen et al., 2023; Zhang et al., 2024). These three crops are commonly grown in local areas, particularly in mining regions and neighboring areas, and serve as significant commercial and food crops. By comparing the physical and chemical properties of these crops in mining and non-mining soils, we can uncover the direct effects of soil pollution on crop growth in mining areas. Furthermore, by analyzing the soil physicochemical properties in conjunction with the diversity and potential functions of these three crop root endophytic bacteria across various soil environments, we can gain insight into how microbial communities in crop roots are influenced by the soil environment in mining areas.

This study aims to reveal the impact mechanism of soil contamination in mining areas on the microbial community structure of crop roots by comparatively analyzing the physicochemical properties of soil and the diversity of endophytic bacteria in the roots of *Musa basjoo*, *Amygdalus persica*, and *Triticum aestivum* in both mining and non-mining areas. This study explored the effects of abandoned iron ore mine soil on endophytic bacteria in the root systems of local cash crops and food crops, providing a scientific basis for strain selection and breeding for crop improvement in mining areas, sustainable development of agriculture and food safety guarantee.

## Materials and methods

### Study locations and sample collection

The experimental site was located in Wuding County, Chuxiong Yi Autonomous Prefecture, Yunnan Province (101°55′-102°29′E, 25°20′-26°11′N). The proven minerals in terms of geological structure consist of metals like iron, titanium, copper, lead, and zinc, along with non-metallic minerals like phosphorus and gypsum. During April 2024, we systematically collected three crops that are widely planted in the abandoned iron ore area, *Musa basjoo Siebold*, *Amygdalus persica*, and *Triticum aestivum*. The three crops in the mining area were named Mbs, Ape, and Tae. In a non-mining area adjacent to the mining area with similar environmental conditions, soil and root tissues of the same three crops were collected as a control group. The three crops in the non-mining area were named Mbs-CK, Ape-CK, and Tae-CK, respectively. Adhering to the strict 5-point sampling method (Data collection was carried out by selecting five equally spaced sample points within a given area.), three independent sampling points were placed in each designated area to ensure the samples’ representativeness. While conducting the sampling, the soil layer within 0 to 5 cm depth was extracted to obtain rhizosphere soil and plant roots (The root systems of most plants are usually in the 0–5 cm depth range, and these root systems are the primary interface for plant–soil microbial interactions.). Following the removal of loose soil around the roots, the soil remaining on the roots was collected using a sterile brush and identified as rhizosphere soil. Roots, leaf litter and stones have been removed from inter-root soil samples where they could affect the results of the analyzes (Zhao et al., 2024). Afterwards, the soil was filtered through a 2 mm sieve and air-dried for a series of physicochemical property analyzes to comprehensively understand the soil quality. The samples were stored at −20°C.

### Measurement of soil physicochemical properties

The pH of the soil was measured precisely using glass electrode technology (NY/T 1121.2-2006). The organic carbon content in the soil was quantitatively analyzed through the oxidation of potassium dichromate, combined with external heating (Shen et al., 2024). Soil total nitrogen was determined through the pretreatment step of combined digestion with sulfuric acid and an accelerator, followed by the Kjeldahl method (LY/T 1228-2015). The determination of soil total phosphorus was performed through NaOH alkali fusion treatment, followed by quantitative analysis using molybdenum-antimony spectrophotometry. The total potassium content was determined through NaOH melt flame spectrometry (Wang et al., 2024). The measurement of soil alkaline nitrogen was based on the principle of alkaline hydrolysis diffusion (LY/T 1229-1999). Soil effective phosphorus is determined by combined leaching of ammonium fluoride-hydrochloric acid solution and sodium bicarbonate solution, followed by molybdenum antimony antimony colourimetric method to complete the determination of its content (NY/T 1121.7-2014). The multi-element contents of the soil were determined via four-acid digestion, inductively coupled plasma-mass spectrometry (DZT 0279.2-2016). The measurement of each soil sample was conducted at least three times, and consequently, the mean value was derived.

### DNA extraction, sequencing, and analysis from root tissues

In order to obtain pure samples of plant root microorganisms, the surface soil was first washed with sterile distilled water, soaked in 70% ethanol, and then washed several times with sterile distilled water until the washings were free of colony growth after incubation on culture medium. Genomic DNA was extracted using the SDS method. The bacterial V5–V7 highly variable region of the 16S rRNA gene was amplified using genomic DNA diluted to 1 ng/μl with sterile water as a template, with primers 799F (5′-AACMGGATTAGATACCCKG-3′) and 1193R (5′-ACGTCATCCCCACCTTCC-3′). The library was constructed using NEBNext^®^ Ultra^™^ IIDNA Library Prep Kit, the constructed library was subjected to Qubit and Q-PCR quantification, and after the library was qualified, NovaSeq6000 was used for on-line sequencing.

The sample data were truncated to remove the Barcode and primer sequences and then the reads of each sample were spliced using FLASH to obtain Raw Tags (Magoč and Salzberg, 2011). The spliced Raw Tags were strictly filtered using fastp software to obtain Clean Tags (Bokulich et al., 2013; Deng et al., 2024). The above processed Tags were compared with the species annotation database and the chimeric sequences were removed to obtain the final Effective Tags (Edgar et al., 2011). For the Effective Tags, the DADA2 module of QIIME2 (Version QIIME2-202006) software was used to reduce noise and filter out the sequences with abundance less than 5 to obtain the final ASVs. The ASVs obtained were then compared to a database using the classify-sklearn module in the QIIME2 software in order to identify the species corresponding to each ASV (Wang et al., 2021). Tables of species abundance at different taxonomic levels were obtained based on the results of the ASVs annotation, and statistical tests of differential abundance were performed (Gloor et al., 2017).

### Alpha analysis, beta analysis, and function prediction

To analyze the microbial community diversity within the samples, alpha diversity indices (observed_otus, shannon, simpson, chao1, goods_coverage, and pielou_e) were calculated for the different samples using QIIME2 software and visualized using R v2.15.3. For the comparison of microbial community composition among different samples, Beta Diversity Index intergroup difference analysis was used. This method detects differences between different samples (groups) using Principal Coordinates Analysis (PCoA). For the functional prediction of different microorganisms, metagenomic functional prediction was carried out in the COG, KO, and pathway databases based on marker genes (16S rRNA) using PICRUSt2 software. Correlation heatmaps were used to show the correlations between inter-root soil physicochemical properties and alpha diversity indices, CCA and RDA was used for correlation analysis between soil physicochemical properties and root endophytic bacterial microbiota. The RDA analysis was performed by the genescloud tools.

### Statistical analysis

Excel 2019 was used to calculate the diversity indices, such as each soil physicochemical index. Analysis of variance (ANOVA) was performed using SPSS 21.0 statistical software. Correlation analysis of significantly different bacteria based on 16S rRNA analysis was performed according to the Pearson correlation coefficient. The data satisfies the normal distribution based on the Anderson-Darling test and meets the homogeneity of variances test when analyzed using SPSS. The significance of differences between samples was analyzed: a *t*-test was used for two groups of samples, and Tukey’s test was used for more than two groups of samples. By employing Duncan’s multiple range test, distinctions among different means were established at a significance level of *p* < 0.05.

## Results

### Analysis of soil trace elements and physicochemical properties

The values of the soil pH, OC content and trace element contents of Fe, Zn, Cu, Ti and Pb in the mining area test group and non-mining control group are shown in Table 1. The pH values of the Ape and Tae experimental groups both decreased significantly (*p* < 0.05). This change was not significant between the acidic soil control Mbs-CK and the Mbs experimental groups. The pH value of the Mbs group was lower than that in all the experiments, and it was the lowest in the group. Concerning the OC content, each of the experimental groups demonstrated a progressive trend when compared to their corresponding control groups, with the Mbs experimental group showing the most substantial increase of 212%. The mining of iron ore has resulted in a generally higher Fe content in the soil of the experimental group compared to the control group, while the Ti levels were lower in the experimental group compared to the control group. Additionally, the Ape and Tae experimental groups exhibited significantly lower levels of Zn and Cu compared to the control groups (*p* < 0.05). On the other hand, the levels of Pb in the Mbs and Ape experimental groups were observed to be elevated compared to the control group.

**Table 1:** Inter-root soil pH and basic element contents of three crops in mining and non-mining areas.

Groups	Mbs	Mbs-CK	Ape	Ape-CK	Tae	Tae-CK
pH	5.67 ± 0.03e	5.30 ± 0.02f	5.79 ± 0.01d	7.55 ± 0.02a	6.01 ± 0.01c	7.42 ± 0.02b
Organic carbon (g/kg)	44.60 ± 1.37e	14.28 ± 0.19d	79.73 ± 1.42a	66.86 ± 0.74b	51.56 ± 0.79c	46.24 ± 0.38d
Fe (g/kg)	72.55 ± 3.69a	48.18 ± 1.16c	68.64 ± 2.52a	42.43 ± 1.54d	70.54 ± 2.31a	56.04 ± 1.61b
Zn (mg/kg)	55.15 ± 2.44d	29.33 ± 1.07e	63.45 ± 2.10c	215.41 ± 10.06a	48.37 ± 1.20d	82.20 ± 2.76b
Cu (mg/kg)	52.81 ± 2.21c	21.14 ± 0.88d	65.11 ± 0.98b	184.36 ± 9.38a	20.18 ± 1.01d	69.04 ± 3.44b
Ti (g/kg)	4.75 ± 0.26c	5.23 ± 0.18b	3.92 ± 0.11d	6.21 ± 0.26a	4.40 ± 0.21c	5.19 ± 0.19b
Pb (mg/kg)	50.01 ± 1.73a	40.23 ± 1.72b	47.29 ± 2.14a	29.20 ± 1.52d	33.20 ± 1.01c	49.14 ± 2.07a

Different letters represent significant differences between treatments (*p* < 0.05).

**Figure 1:**
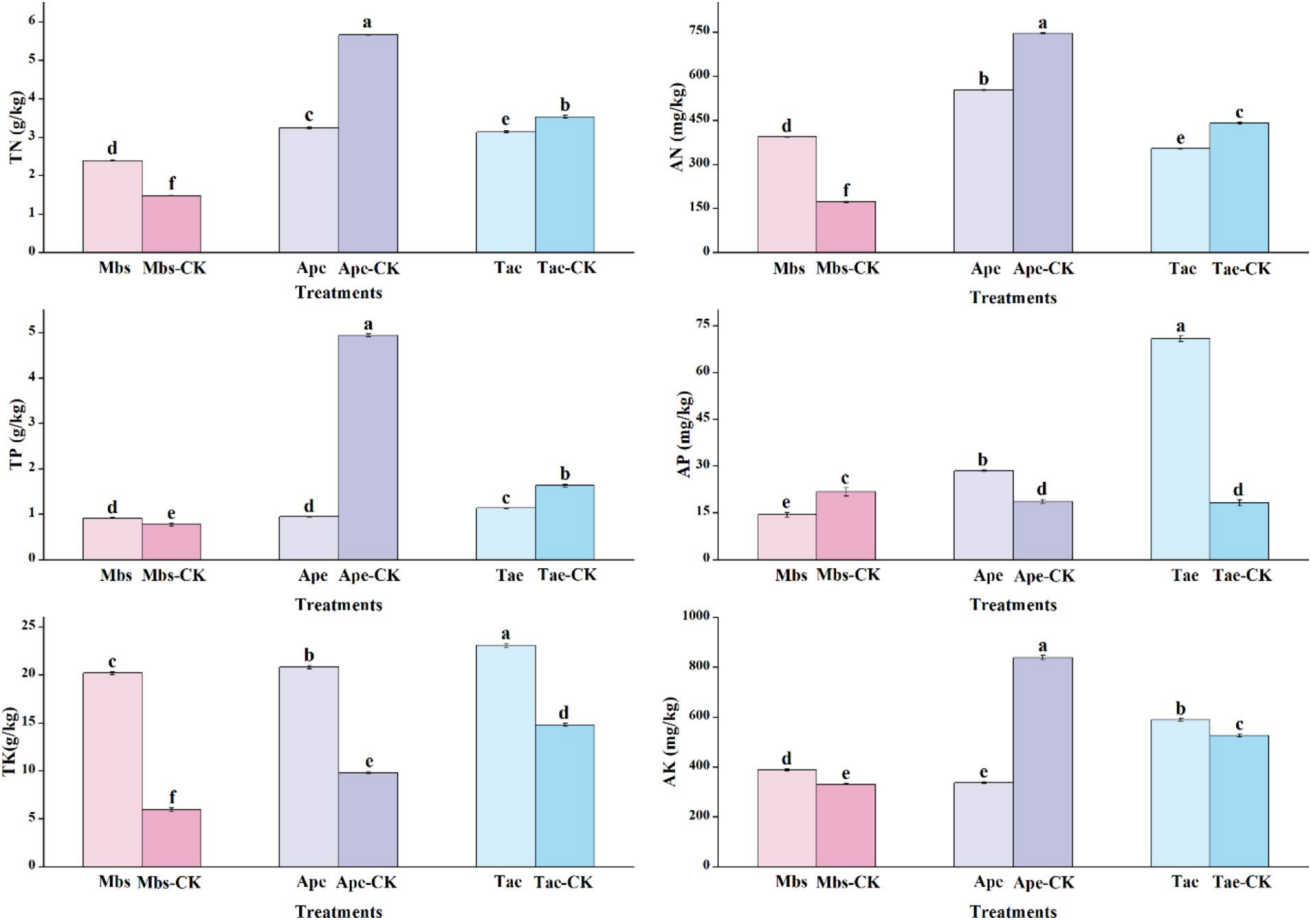
Physico-chemical properties of inter-root soils of three crops in mining and non-mining areas. Mbs, Ape, and Tae were mining test groups for *Musa basjoo Siebold*, *Amygdalus persica*, and *Triticum aestivum*, respectively. Mbs-CK, Ape-CK, and Tae-CK were non-mining blank controls for each of the three crops. Soil total nitrogen (TN), alkaline nitrogen (AN); total potassium (TK), effective potassium (AK), total phosphorus (TP), effective phosphorus (AP) (different letters represent significant differences between treatments) (*p* < 0.05).

In different groups, we also measured the total nitrogen (TN), alkaline nitrogen (AN), total kilocalories (TK), available kilocalories (AK), total phosphorus (TP), and available phosphorus (AP) of the soil in different environments, as well as other indicators of soil physicochemical properties (Figure 1). The soil physicochemical properties (*p* < 0.05) showed significant differences between the experimental group and the control group across the three plants. The TN and AN contents in the mining area under the Mbs system were significantly higher than those in the non-mining control group in terms of nitrogen. Despite this, the nitrogen content in the mining soil where the Ape and Tae crops were planted was considerably lower than in the control group, particularly in the case of the Ape.

The phosphorus levels showed contrasting patterns of change. While the TP content in the soil of the mining area under Mbs was high, its AP content was significantly lower than that of the control group. In contrast, the AP content in the soil of the mining area under Ape and Tae was notably higher than that in the control group, especially under Tae. The AP content increased by 289%, particularly under Tae. Notably, the TP content in the Ape soils significantly decreased to one-fourth of that in the control group. The soil in the experimental groups across all mining areas showed a significantly higher TK content compared to the control group, indicating a richness of potassium in the mining area or an increase in potassium input due to mining activities. However, for AK, the soil content in the mining area under Mbs and Tae outperformed the control group, whereas Ape fell short compared to the control group.

In general, the mining environment makes the soil nutrient content complex and variable. Different crops have different growth characteristics under mining area conditions. Crops maintain a relatively stable internal environment by increasing or decreasing the uptake of elements. The pH value of the soil in the mining area was generally lower and weakly acidic. The contents of OC, Fe, and TK were higher than those in the non-mining area, while the Ti content was lower. The contents of other elements varied due to the adaptability of different plants. Mbs had an advantage in promoting the accumulation of nitrogen and potassium. Ape faced the challenge of decreasing nitrogen, phosphorus, and available potassium contents. Tae significantly activated phosphorus, giving it an advantage.

### High-throughput sequencing data analysis and microbial community composition

The ASV data were obtained with noise reduction. The results of the Venn diagram (Supplementary Figure S1) revealed that 3,316 endophytic bacterial ASVs were detected in the roots of the three crops in the mining area test group. Of these, the Mbs, Ape, and Tae values were 1765, 1,011, and 1,120, respectively, and 11 species were common to all three crops. In contrast, 1,332 endophytic bacteria, namely ASV, Mbs-CK, Ape-CK, and Tae-CK, were detected in the roots of the three crops in the control group in the non-mining area (365, 505, and 584), respectively. This number was lower than that in the experimental group, but 101 endophytic bacteria were shared by the three species. There were 66 types of Meb and Mbs-CK, 41 types of Ape and Ape-CK, and 94 types of Tae and Tae-CK. To further assess the richness of bacterial communities under different crops and treatments, we used the sparse curve of OTUs (Supplementary Figure S2) as an analysis tool. Sparse curve analysis revealed that all of the soil samples had sufficient sequencing depth. When the sequencing read length exceeded 6,000 reads, the dilution curve gradually stabilized, the amount of sequencing data progressed reasonably, and more data volume did not significantly affect the alpha diversity index, ensuring the accuracy and reliability of microbial diversity analysis. Therefore, the abundance of sequences can be used to infer the diversity of bacteria in the samples.

**Figure 2:**
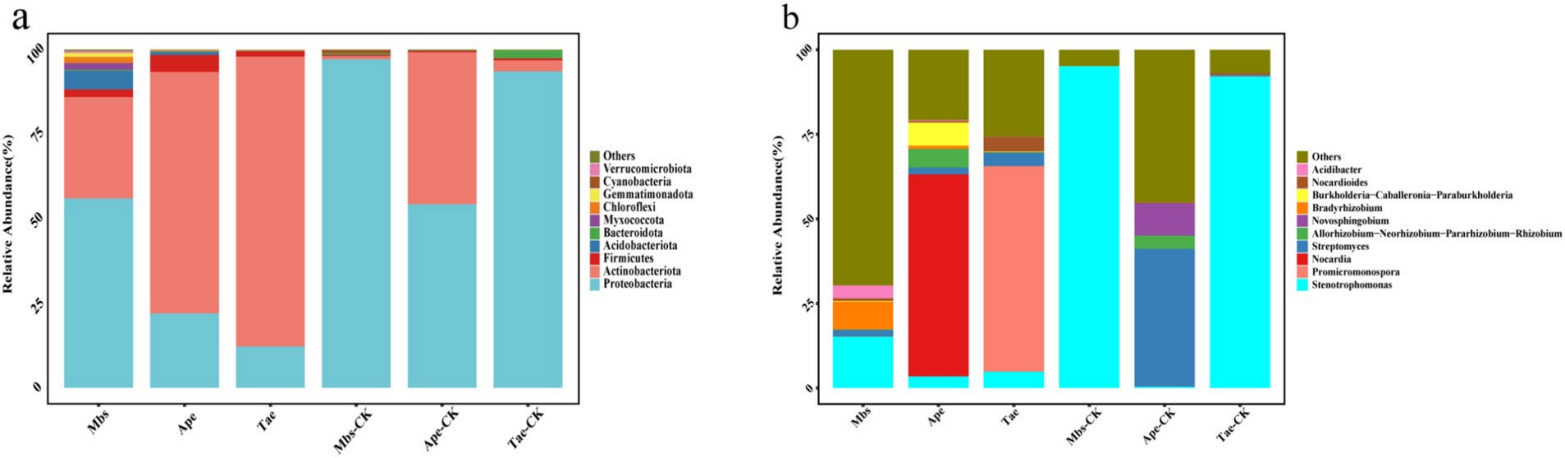
Effect of mining soils on the relative abundance of endophytic bacteria (top 10) at the phylum level **(A)** and genus level **(B)** in three crops. Mbs, Ape, and Tae were mining test groups for *Musa basjoo Siebold*, *Amygdalus persica*, and *Triticum aestivum*, respectively. Mbs-CK, Ape-CK, and Tae-CK were non-mining blank controls for each of the three crops.

The results showed that in all root samples, 23 phyla, 55 classes, 131 orders, 231 families, and 427 genera of endophytic bacteria were identified. We compared the changes in the top 10 most abundant phyla and genera (Figure 2). The main endophytic bacterial taxa at the phylum level were *Proteobacteria*, *Actinobacteria*, *Firmicutes*, *Acidobacteriota*, and *Bacteroidota*, which accounted for more than 90% of the total bacterial abundance. *Proteobacteria* was the most abundant, accounting for 55.88% of all bacteria. All experimental groups exhibited a significantly lower abundance of *Proteobacteria* and a significantly higher abundance of *Actinobacteria* when compared to the control group (*p* < 0.05). At the genus level, *Stenotrophomonas* was the main bacterial group, accounting for 35.2% of all bacteria. In comparison to the control group, the Mbs and Tae groups showed significantly lower abundance of *Stenotrophomonas*. Moreover, the Ape group exhibited significantly lower abundance of *Streptomyces*, while the Mbs group had significantly lower abundance of *Bradyrhizobium*. The abundance of *Nocardia* in the Ape group increased by 59.69%, and the abundance of *Promicromonospora* in the Tae group increased by 60.72%.

### Analysis of the microbial diversity of the endophytic bacteria in the root system

**Figure 3:**
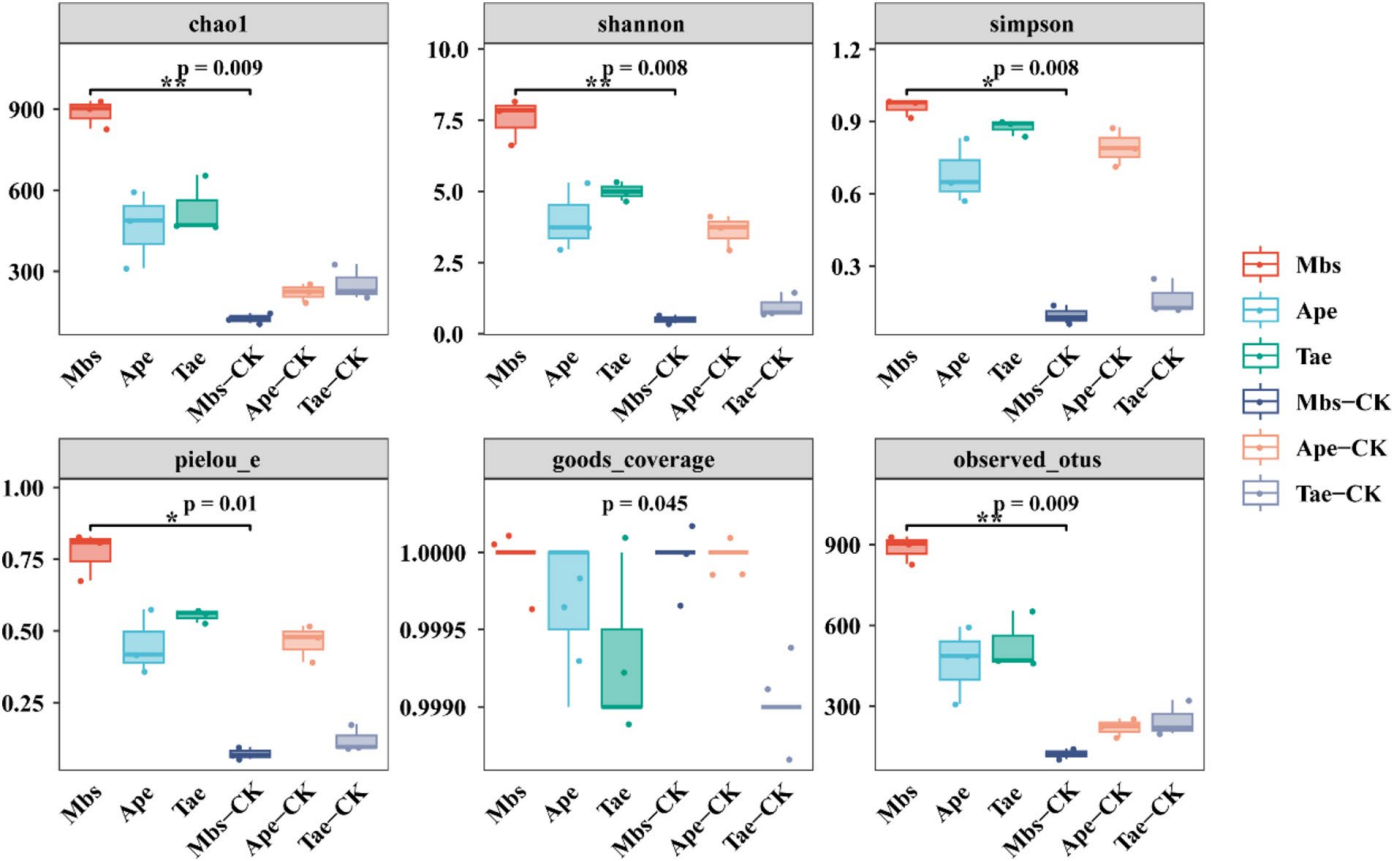
Alpha diversity indices of endophytic bacterial communities of three crops in mining and non-mining areas. Mbs, Ape, and Tae were mining test groups for *Musa basjoo Siebold*, *Amygdalus persica*, and *Triticum aestivum*, respectively. Mbs-CK, Ape-CK, and Tae-CK were non-mining blank controls for each of the three crops (*p* < 0.05).

We calculated the α-diversity indices of the three crops in the experimental group and the non-mining control group (Figure 3). Compared with the control group in the non-mining area, all the experimental groups showed increased Chao1 and Shannon indices (Supplementary Figure S3). Compared with Mbs-CK, Mbs also significantly increased the Simpson, Pielou_e, and observed OTUs indices (*p* < 0.05), indicating that the community diversity was greater and the species distribution was more even. The Simpson diversity of Tae significantly increased by 71.3% (*p* < 0.05) compared to Tae-CK, while the Simpson diversity of the Ape test group did not significantly change.

In order to delve deeper into the beta diversity among the flora in various regions and distinguish the variations in microbial community structure, we conducted PCoA in conjunction with OTU abundance using the weighted UniFrac distance (Supplementary Figure S4). The PC1 and PC2 axes explained 55.36 and 25.45% of the results, respectively. The distance separating Mbs-CK and Tae-CK was relatively near, unlike the other samples, while the distance between Ape and Ape-CK was also close, indicating a greater overlap in bacterial communities.

### Functional prediction of endophytic bacteria in the root system

**Figure 4:**
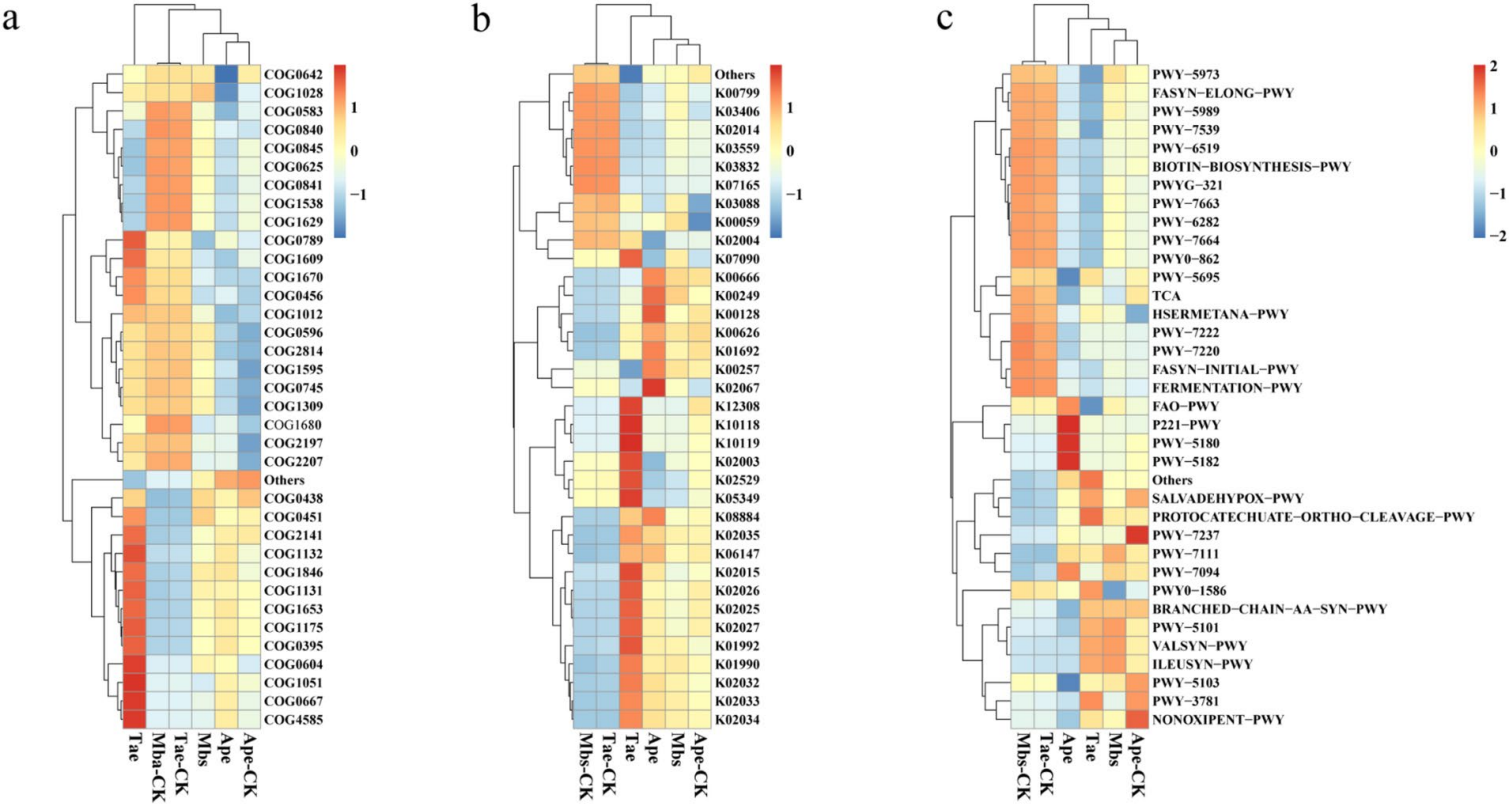
Functional prediction of three crop endophytic bacteria based on the COG **(A)**, KO **(B)**, and pathway **(C)** databases of PICRUSt2. Mbs, Ape, and Tae were mining test groups for *Musa basjoo Siebold*, *Amygdalus persica*, and *Triticum aestivum*, respectively. Mbs-CK, Ape-CK, and Tae-CK were non-mining blank controls for each of the three crops.

For our experiments, we utilized PICRUSt2 to forecast bacterial functions. The top 35 functions were selected based on their abundance and functional annotation information in the COG, KO, and pathway databases. These functions were then depicted in a heat map along with their abundance information for each sample. They were then clustered at different functional levels (Figure 4). According to the COG database, the experimental group promoted the functions of flavin-dependent oxidoreductase (COG2141) and DNA-binding transcriptional regulator (COG1846). Compared to the control group, the Mbs experimental group showed increased functions of nucleoside-diphosphate-sugar epimerase (COG0451) and NAD(P)-dependent dehydrogenase (COG1028), and decreased functions of DNA-binding transcriptional regulator (COG0789). In comparison to Ape-CK, Ape reduced the function of signal transduction histidine kinase (COG0642). In contrast, the Tae group showed increased functions of signal transduction histidine kinase ComP (COG4585), pyridoxal reductase PdxI or related oxidoreductase (COG0667), ADP-ribose pyrophosphatase YjhB (COG1051), and DNA-binding transcriptional regulator, MerR family (COG0789) genes when compared to the control group.

In the KO database, the Myb_DNA-binding (K00249) function of Mbs was upregulated compared to that of the control. Compared to the control, Ape increased the ability of light to harvest chlorophyll a/b-binding protein (K02067). Tae increased the expression levels of raffinose transport system permease protein (K10119) and beta-galactosidase (K12308) compared to those in the control group.

In the pathway database, the function of pyruvate fermentation to isobutanol (PWY-7111) was upregulated in the experimental group. Compared to the control group, the function of L-isoleucine biosynthesis I (ILEUSYN-PWY) in Mbs was upregulated, while the function of peptidoglycan maturation (PWY0-1586) was downregulated. Additionally, the function of toluene degradation II (PWY-5182) was upregulated, while the function of urate biosynthesis (PWY-5695) was downregulated. Furthermore, Tae decreased the function of fatty acid β-oxidation I (FAO-PWY) compared to the control group.

### Correlation analysis

**Figure 5:**
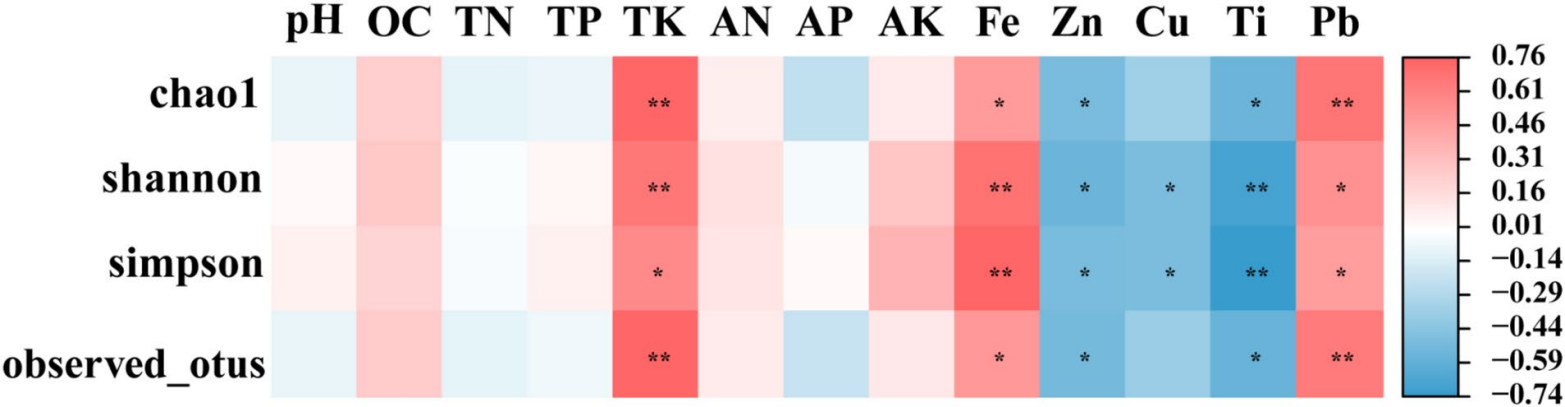
Correlation between physical and chemical properties of inter-root soil and alpha diversity index of endophytic bacteria. Mbs, Ape, and Tae were mining test groups for *Musa basjoo Siebold*, *Amygdalus persica*, and *Triticum aestivum*, respectively. Mbs-CK, Ape-CK, and Tae-CK were non-mining blank controls for each of the three crops. * represents significant, ** represents and its significant (*p* < 0.05).

To investigate the associations between the soil physicochemical parameters and the α diversity indices of the root endophytic bacteria, Pearson correlation analysis (PC, *p* < 0.05) was conducted (Figure 5). Results indicated a significant positive correlation between the soil TK content, as well as the Fe and Pb metal contents, and the α diversity index. Conversely, the α diversity index exhibited a significant negative correlation with the Zn and Ti contents. Additionally, the Shannon and Simpson indices were negatively correlated with the Cu content (*p* < 0.05).

**Figure 6:**
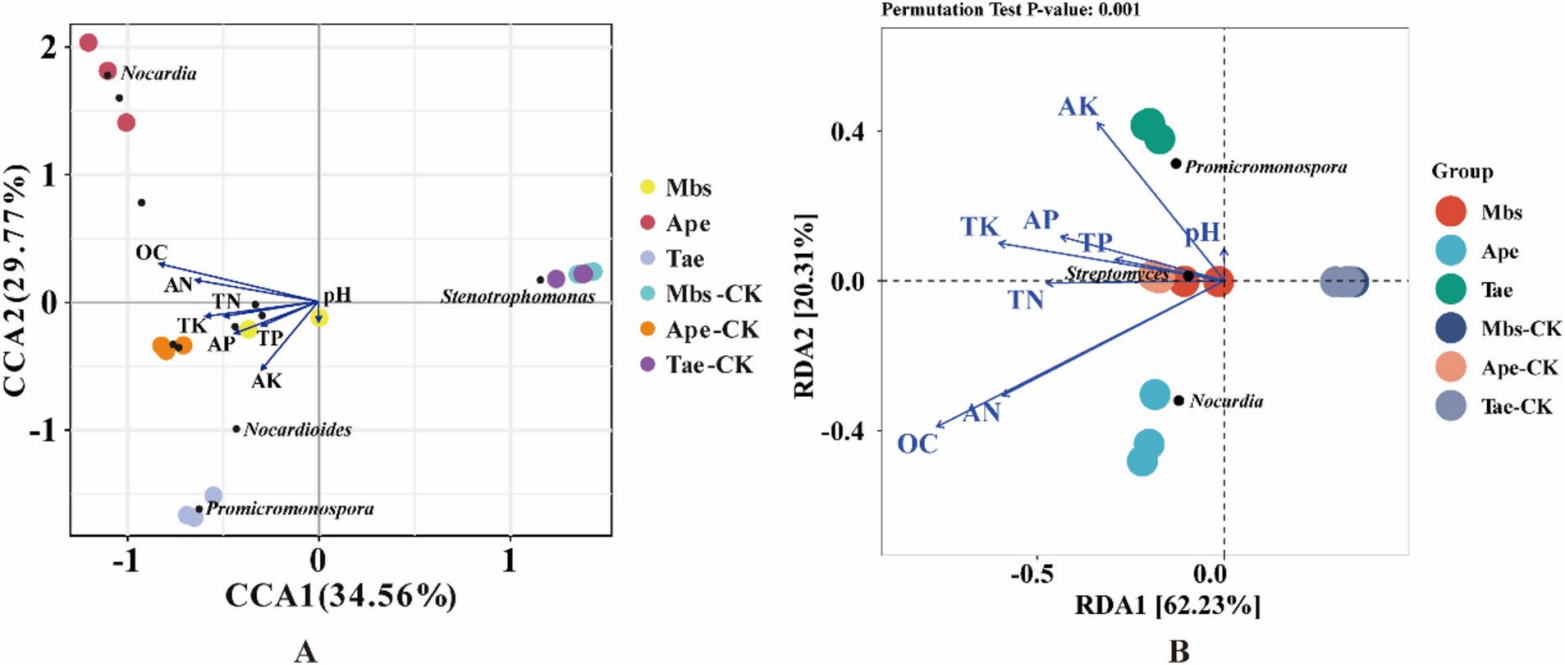
CCA **(A)** and RDA **(B)** of microbiota of root endophytic bacteria associated with soil physicochemical factors. Mbs, Ape, and Tae were mining test groups for *Musa basjoo Siebold*, *Amygdalus persica*, and *Triticum aestivum*, respectively. Mbs-CK, Ape-CK, and Tae-CK were non-mining blank controls for each of the three crops. The blue arrows represent the different influencing factors, and the angle between the influencing factors represents the magnitude of the correlation between them; an acute angle indicates that the two factors are positively correlated, a right angle is not correlated, and a negative correlation occurs at an obtuse angle, and the longer the rays are, the greater the role of the factor in influencing the composition/functioning of the bacterial colony.

In order to better understand how environmental factors impact the microflora of root soil and endophytic bacteria in root systems, we conducted redundancy analysis. Our focus was on the top 10 most prevalent microbial species at the genus level and how they relate to soil environmental factors (Figure 6). RDA 1 and RDA 2 explained 62.23 and 20.31%, respectively, of the total variation, revealing the complex connection between environmental factors and the composition of microflora. Among them, *Promicromonospora* was significantly positively correlated with the soil AK content, *Nocardia* was significantly positively correlated with the soil AN and OC contents, and *Streptomyces* was significantly positively correlated with the soil TK, TP, and AP contents.

## Discussion

Mining disturbances can degrade land and change the soil nutrient content (Xiao et al., 2021; Wang et al., 2023). Our findings showed that the community composition of endophytic bacteria in the root system was significantly impacted by the diverse soil physicochemical properties of the mining area and non-mining area. Soil with a pH close to neutral had a greater variety of beneficial microorganisms. The soil samples from the three crops collected in the mining area were all weakly acidic (Li C. et al., 2022). In terms of the OC content, the soil in the experimental group accumulated more organic carbon, with a particularly prominent effect on Mbs. The differences in trace element contents may indicate that these treatments promoted the accumulation of Pb in the soil or reduced its loss to some extent. Enhancements in the microenvironment could potentially alter the microbial community composition, leading to a higher prevalence of bacteria, which play a crucial role in driving N transformation (Liu et al., 2018). Compared to Mbs-CK, the contents of AN and TN in the rhizosphere soil of Mbs increased significantly. Previous studies have found that a large amount of heavy metal dust is blown away and dispersed through the air in the vicinity of mining sites (Kicińska and Wikar, 2021). We speculate that during the process of iron ore mining, some nitrogen-containing wastes or pollutants may be generated, which could potentially enter the soil through pathways such as rainfall runoff and wind deposition, thereby increasing the nitrogen content in the soil. However, the values of Ape and Tae were significantly lower than those of the control group. This may enhance the uptake or retention of nitrogen in the mining environment, maintaining the stability of the internal environment (Chen et al., 2024). Compared to the control group, the Mbs had a higher TP content and lower AP content. This could indicate that the efficiency of phosphorus transformation in the soil is reduced, or that phosphorus is being fixed. The opposite was true for the Ape and Tae, which may be related to the activation of phosphorus or the activities of specific microorganisms by these two crops in the mining environment. Notably, the TP content in the soil of the Ape significantly decreased to one-fourth of the control level. The difference in composition also affects the Shannon diversity of bacteria (Yuan H. et al., 2024). Pb can inhibit normal plant function, and it has been shown that *Arabis alpina* L. can transfer Pb from the root system to the ground (Li et al., 2019). In our study, the Pb content of both the Mbs and Ape soils was significantly greater than that of the CK soil, while that of the Tae was the opposite, indicating that the roots of Tae may have a greater Pb content than the CK level. *Triticum aestivum* is highly sensitive to Pb, and differences in soil properties such as pH and organic matter content affect Pb transfer in the soil-wheat system (Wu et al., 2020).

The plant root system serves as the home for a diverse range of microorganisms, which are essential for both disease progression and plant growth (Rotoni et al., 2022). The composition of microbial communities is intricately connected to soil physicochemical properties. Differences in soil physicochemical properties can affect changes in microbial communities (Wang et al., 2017; Li Q. et al., 2024). Among the phyla of all the samples, the phylum with the highest abundance was *Proteobacteria*, which was a key group after mining disturbance, with a wide range of degradation and metabolism. *Proteobacteria* are predominantly characterized by their capacity to flourish in highly unstable surroundings, which in turn enables them to uphold their normal metabolism through their rapid growth (Zeng et al., 2017; Fei et al., 2024). *Actinomycetes* also account for a relatively high proportion. They play an important role in vegetation succession and may cause changes in the ecological functions of mining areas and non-mining areas. Many studies have shown that they constitute the main microbial community in mining-contaminated areas (Morrissey et al., 2017; Jin et al., 2022; Pan et al., 2022). The changes in bacteria at the genus level varied among the different crops. Among the genera of all the samples, *Stenotrophomonas* accounted for the greatest proportion. This genus of bacteria is capable of extracting heavy metals from intricate mining water and is crucial in the ecological rehabilitation of mining regions (Sánchez-Castro et al., 2021). The abundance of *Nocardia* increased in the Ape group. This genus of bacteria can help the host better resist heavy metal stress, increase host resistance and improve repair ability (Jiang et al., 2024). Based on information from three databases, we were able to predict the bacterial community function of endophytic bacteria in various environments to analyze how endophytic bacteria react to mining areas. The findings showed that the mining environment caused an increase in the functions of histidine kinases, oxidoreductases, DNA-binding transcription regulators, and β-galactosidases, aligning with previous research results (Li Q. et al., 2024). Histidine kinases are able to sense changes in the external environment and regulate the expression of relevant genes through the signal transduction pathway, thus enabling bacteria to survive and reproduce in different environments, and are an important component of plant growth and development (Gupta et al., 2020). Oxidoreductases are a class of enzymes that catalyze oxidation–reduction reactions, and they play a significant role in mining environments. Through oxidation–reduction processes, these enzymes can alter the valence states of heavy metals, thereby affecting their solubility and mobility (Ding et al., 2024). DNA-binding transcription regulatory factors are key proteins that regulate gene expression. They can bind to DNA and control the transcriptional activity of specific genes. In endophytic bacteria within plants, these regulatory factors may participate in regulating the expression of genes responsive to environmental stressors in mining areas, thereby assisting the bacteria in adapting to the mining environment (Zhou et al., 2024). β-Galactosidase plays a crucial role in the metabolism of cell walls (Niu et al., 2024). In mining environments, β-galactosidase may be involved in processes such as bacterial utilization of carbon sources, cell wall synthesis, and repair, which are significant for the survival and reproduction of bacteria. The enrichment of these bacterial functions indicates that the ability of endophytic bacteria in plants to perceive and respond to stress factors in mining environments has been enhanced. They maintain physiological stability under adverse conditions and may promote interactions between bacteria and plants, thereby aiding plants in maintaining growth and reproduction in unfavorable environments.

Mining activities can cause the microbial composition to vary greatly, affecting the community structure (Liu et al., 2023a). When comparing the α diversity indices of the three crops in different environments, the results showed that the Chao1 index and the Shannon diversity index were significantly higher in the experimental group than in the control group. This trend indicates that the mining area environment has a great impact on these three crops. The species richness and community diversity of the two crops had a positive promoting effect. Under the stress of heavy metal pollution, plants may change the release of active substances, resulting in changes in bacterial communities and affecting the composition and diversity of microorganisms (Yuan X. Q. et al., 2024). Further analysis of the Simpson diversity index revealed that the index was significantly greater in the Mbs and Tae groups than in the control group. This reflects that, in the mining area environment, the distributions of the dominant species in the communities of these two crops were more balanced, and the species diversity was greater. However, the Ape group did not significantly differ from the control group in terms of Simpson diversity. In addition, Mbs presented the highest level of community richness among all the test groups, which further emphasizes the strong adaptability and biodiversity maintenance ability of Mbs in the mining area environment.

This study only explored the relationship between soil physicochemical properties and endophytic bacteria in mine-contaminated soil, without fully investigating the periodic fluctuations of natural conditions such as temperature, light, and precipitation, as well as other unmeasured factors like root parameters. To address these potential limitations, future research can include the establishment of seasonal monitoring points to collect data across different seasons. Additionally, further exploration of the relationship between root parameters and endophytic bacteria, and how they jointly affect crop growth and stress resistance, is necessary. This will help us gain a more comprehensive understanding of the role of endophytic bacteria in crop growth and soil ecosystems.

## Conclusion

This study investigated the physicochemical properties of rhizosphere soil and the diversity of endophytic bacteria in the roots of three plant species grown in mining and non-mining areas. Intriguingly, the diversity of endophytic bacteria in the roots of crops grown in mining areas was found to be increased. In mining environments, the soil pH tends to be weakly acidic, and the content of metal elements is relatively high. Under the stress of pollution, plants undergo changes in their root bacterial communities and functions toward degradation and metabolism to maintain a relatively stable internal environment. The increase in diversity and functional changes support the crops’ adaptation to new environmental conditions. The findings of this study provide data support for the complexity of soil ecosystems in mining areas and plant-microbe interactions, offering a scientific basis for the formulation of ecological restoration strategies and plant cultivation plans in mining areas.

## Data Availability

The original contributions presented in the study are included in the article/Supplementary material, further inquiries can be directed to the corresponding authors.
