# Supplementary material for: The impact of abandoned iron ore on the endophytic bacterial communities and functions in the root systems of three major crops in the local area
Source: Front Microbiol. 2025 Jan 21;16:1536083. doi: 10.3389/fmicb.2025.1536083 (PMC11790582; doi:10.3389/fmicb.2025.1536083)
Supplement: Supplementary file 1 [file Data_Sheet_1.docx]

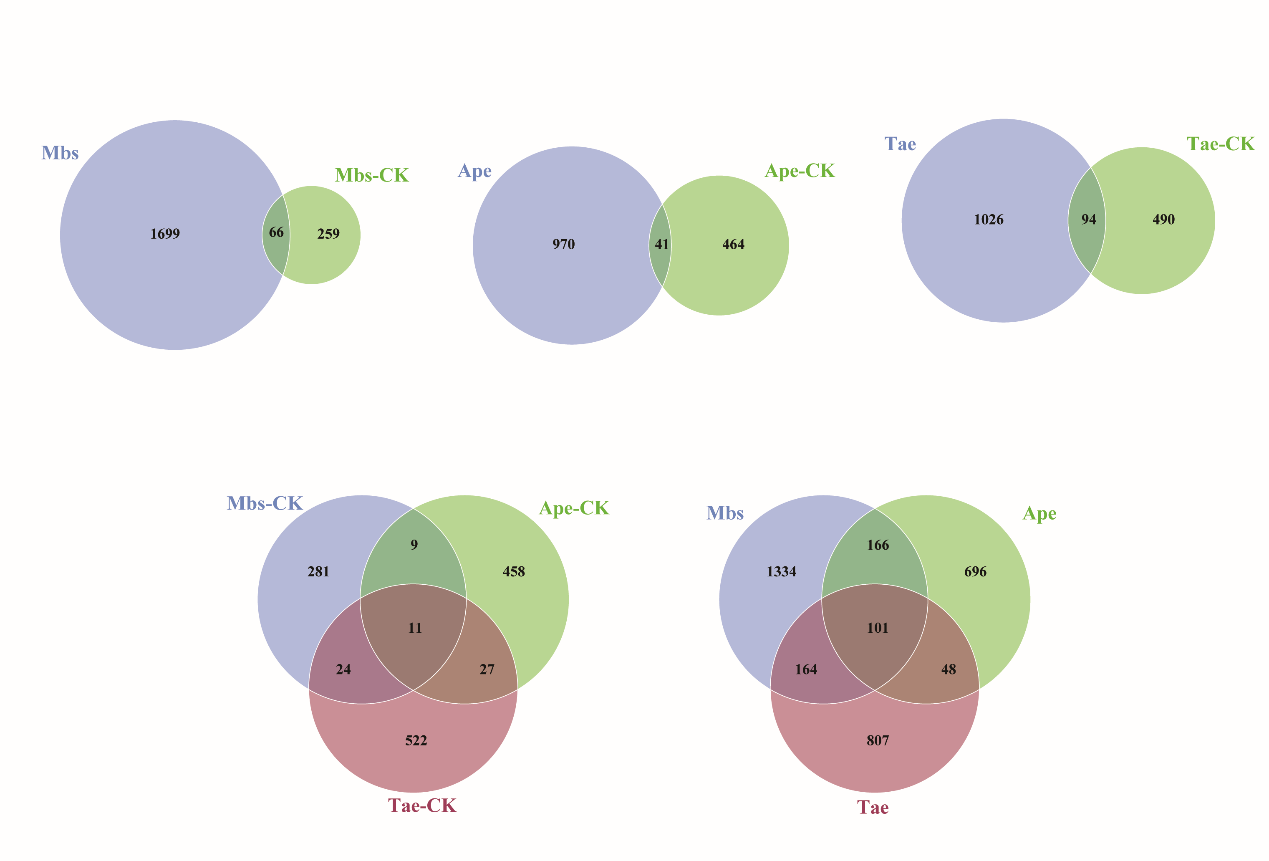


**Figure S 1 Shared and unique OTUs between different samples. (Mbs, Ape, and Tae were mining test groups for *Musa basjoo Siebold*, *Amygdalus persica*, and *Triticum aestivum*, respectively. Mbs-CK, Ape-CK, and Tae-CK were non-mining blank controls for each of the three crops.)**


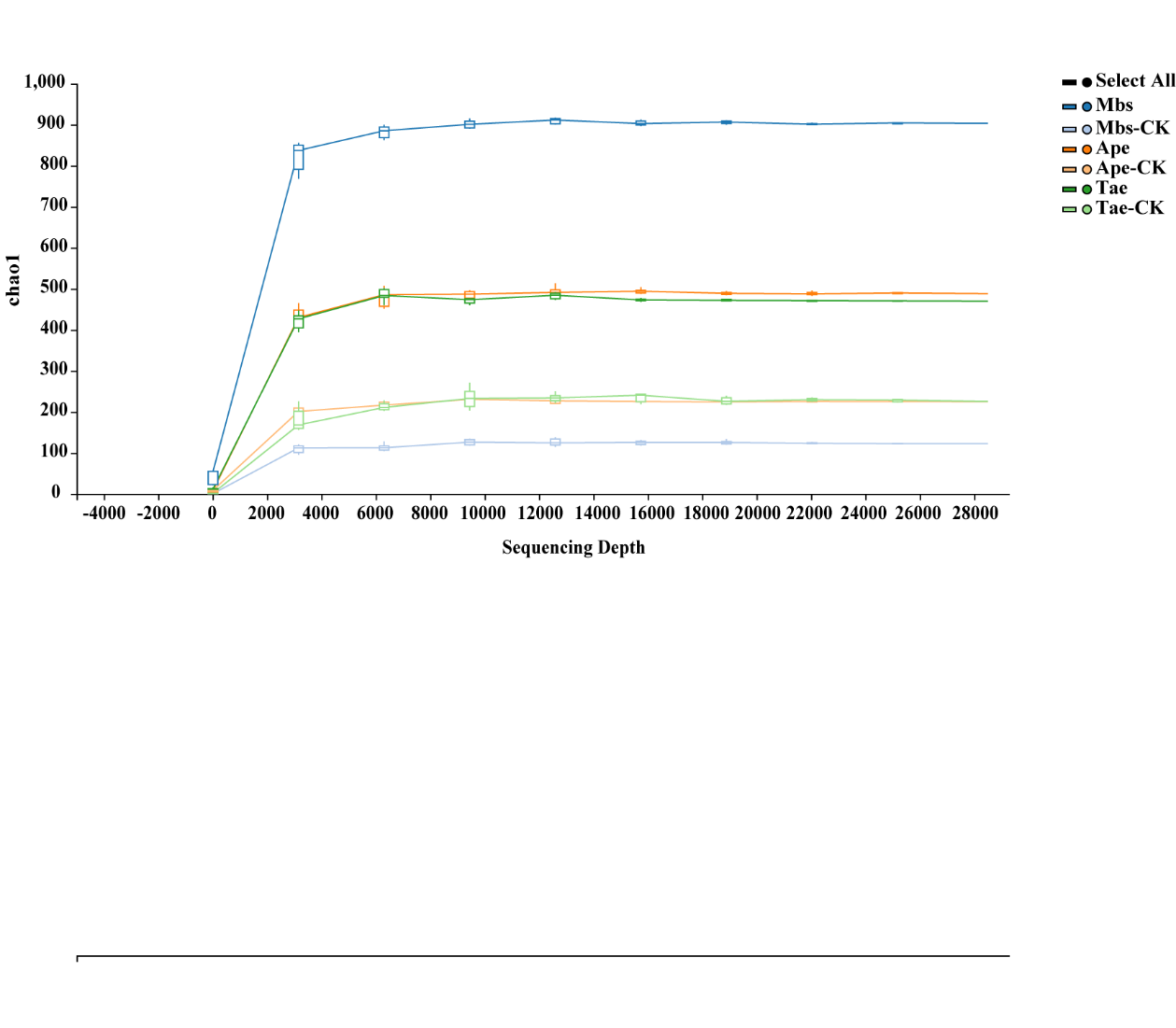


**Figure S 2 Dilution curves of endophytic bacterial species of three crops in mining and non-mining areas. (Mbs, Ape, and Tae were mining test groups for *Musa basjoo Siebold*, *Amygdalus persica*, and *Triticum aestivum*, respectively. Mbs-CK, Ape-CK, and Tae-CK were non-mining blank controls for each of the three crops.)**

**
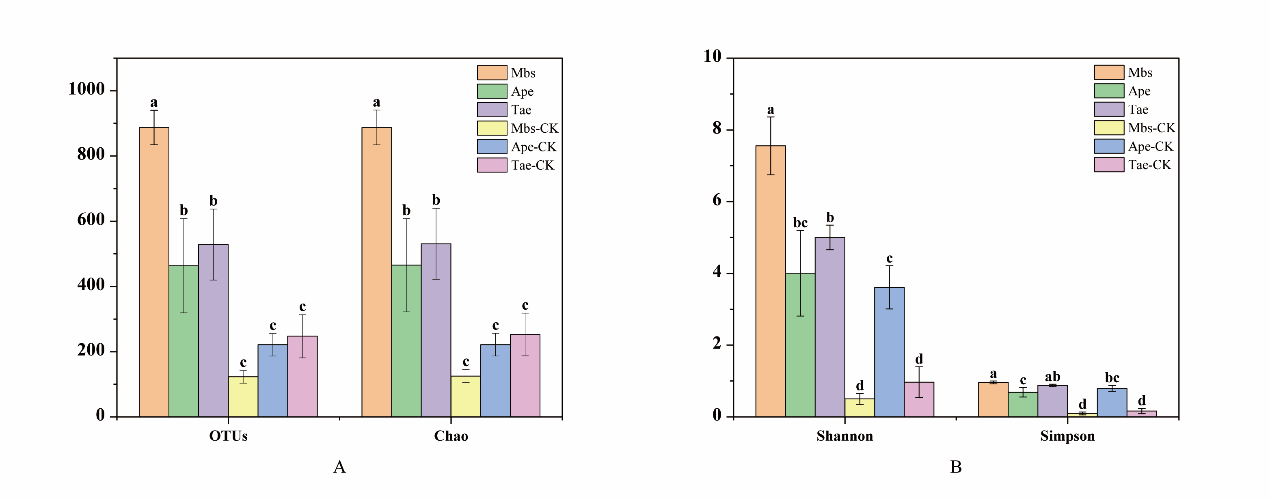
**

**Figure S 3 Histograms of Alpha diversity indices of endophytic bacterial communities of three crops in mining and non-mining areas. (Mbs, Ape, and Tae were mining test groups for *Musa basjoo Siebold*, *Amygdalus persica*, and *Triticum aestivum*, respectively. Mbs-CK, Ape-CK, and Tae-CK were non-mining blank controls for each of the three crops.) (*p*< 0.05)**

**
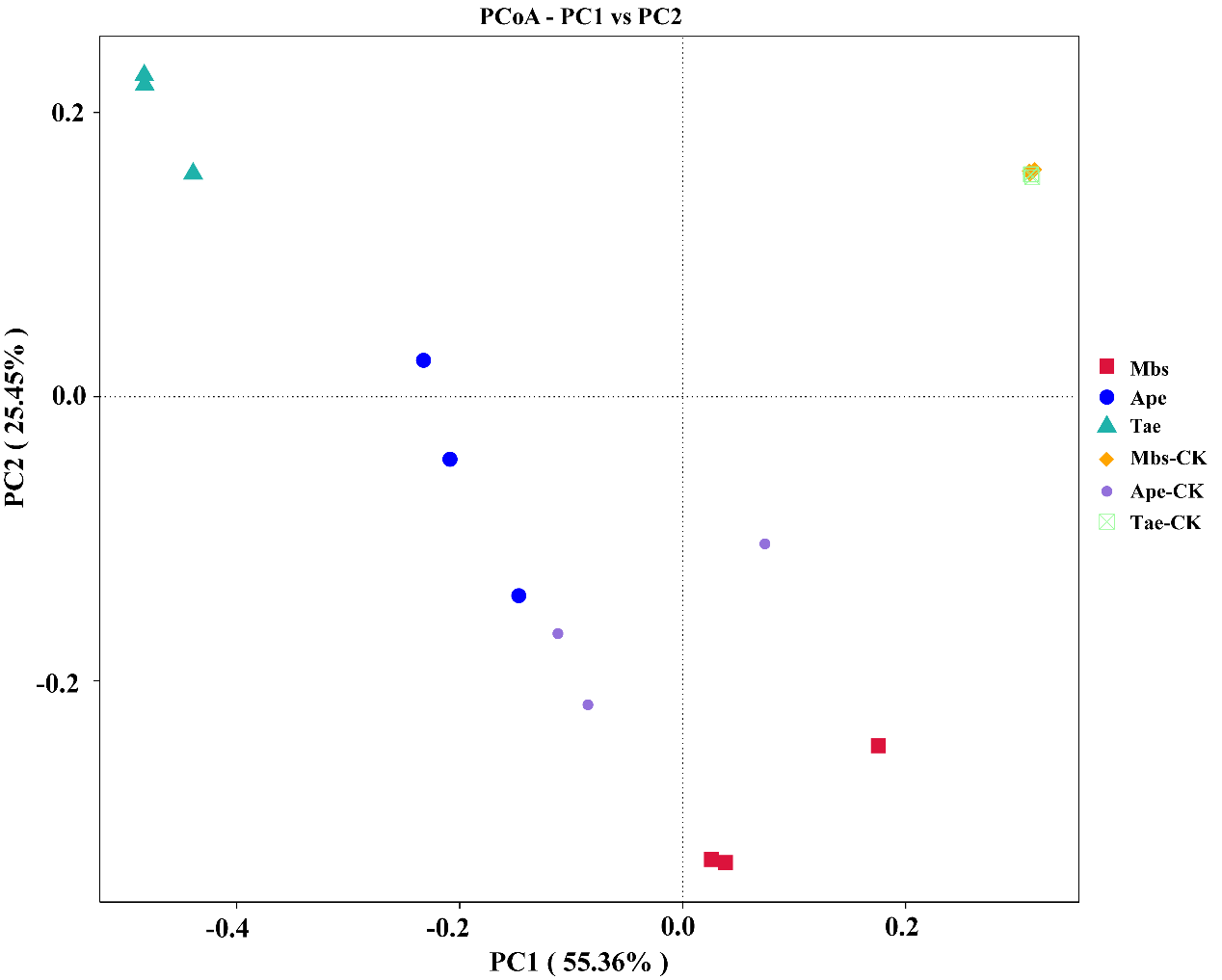
**

**Figure S 4 PCoA analysis based on Weighted Unifrac distances. (Mbs, Ape, and Tae were mining test groups for *Musa basjoo Siebold*, *Amygdalus persica*, and *Triticum aestivum*, respectively. Mbs-CK, Ape-CK, and Tae-CK were non-mining blank controls for each of the three crops.)**
